# Assessment of nutrition and physical activity environments in family child care homes: modification and psychometric testing of the Environment and Policy Assessment and Observation

**DOI:** 10.1186/s12889-017-4686-9

**Published:** 2017-08-29

**Authors:** Amber E. Vaughn, Stephanie Mazzucca, Regan Burney, Truls Østbye, Sara E. Benjamin Neelon, Alison Tovar, Dianne S. Ward

**Affiliations:** 1 Center for Health Promotion and Disease Prevention, University of North Carolina at Chapel Hill, Chapel Hill, USA; 2 Department of Nutrition, Gillings School of Global Public Health, University of North Carolina at Chapel Hill, Chapel Hill, USA; 3 Community and Family Medicine, Duke University School of Medicine, Durham, USA; 4 Department of Health, Behavior and Society, Bloomberg School of Public Health, John Hopkins University, Baltimore, USA; 5 Department of Nutrition and Food Sciences, University of Rhode Island, Kingstown, USA

**Keywords:** Measures development, Reliability, Validity, Young children

## Abstract

**Background:**

Early care and education (ECE) settings play an important role in shaping the nutrition and physical activity habits of young children. Increasing research attention is being directed toward family child care homes (FCCHs) specifically. However, existing measures of child care nutrition and physical activity environments are limited in that they have been created for use with center-based programs and require modification for studies involving FCCHs. This paper describes the modification of the Environment and Policy Assessment and Observation (EPAO) for use in FCCHs.

**Methods:**

The EPAO underwent a through modification process that incorporated an updated format for the data collection instrument, assessment of emerging best practices, tailoring to the FCCH environment, and creation of a new scoring rubric. The new instrument was implemented as part of a larger randomized control trial. To assess inter-rater reliability, observations on 61 different days were performed independently by two data collectors. To assess construct validity, associations between EPAO scores and measures of children’s dietary intake (Healthy Eating Index (HEI) score) and physical activity (accelerometer-measured minutes per hour of moderate to vigorous physical activity, MVPA) were examined.

**Results:**

The modified EPAO assesses 38 nutrition and 27 physical activity best practices, which can be summarized into 7 nutrition-related and 10 physical activity-related environmental sub- scores as well as overall nutrition and overall physical activity scores. There was generally good agreement between data collectors (ICC > 0.60). Reliability was slightly lower for feeding practices and physical activity education and professional development (ICC = 0.56 and 0.22, respectively). Child HEI was significantly correlated with the overall nutrition score (*r* = 0.23), foods provided (*r* = 0.28), beverages provided (*r* = 0.15), nutrition education and professional development (*r* = 0.21), and nutrition policy (*r* = 0.18). Child MVPA was significantly associated with overall time provided for activity (*r* = 0.18) and outdoor playtime (*r* = 0.20). There was also an unexpected negative association between child MVPA and screen time (−0.16) and screen time practices (*r* = −0.21).

**Conclusions:**

The EPAO for the FCCH instrument is a useful tool for researchers working with this unique type of ECE setting. It has undergone rigorous development and testing and appears to have good psychometric properties.

**Trial registration:**

NCT01814215, March 15, 2013.

**Electronic supplementary material:**

The online version of this article (doi:10.1186/s12889-017-4686-9) contains supplementary material, which is available to authorized users.

## Background

Early care and education (ECE) programs are increasingly recognized as important partners in public health initiatives to establish healthy eating and physical activity behaviors in young children [1, 2]. ECE programs can instill healthy habits in children by providing healthy meals and snacks, scheduling time for active play, creating supportive environments, and instituting policies that reinforce good practices. There have been several national efforts in the United States (US) to create recommendations for nutrition and physical activity best practices [3–5]. For example, authorities in child health have published Caring for Our Children, a set of national standards for nutrition and physical activity for ECE programs [3]. Although the attempts to create national standards are informed by current evidence, it has highlighted the need for more ECE-focused research.

Family child care homes (FCCHs) represent the next frontier for ECE research, with a handful of studies already emerging [6–9]. FCCHs are generally smaller programs that operate out of providers’ homes. FCCHs may also be known as “family day care” (in Australia) or “childminders” (in the United Kingdom and across Europe). While these programs have a wide reach, providing care for approximately 1.5 million children in the US [10], they are generally subject to fewer state licensing regulations [11–14]. Observational studies also suggest that children in these settings have poor diet quality [15], low levels of physical activity [16, 17], and higher risk of obesity [18, 19].

Valid and reliable measures of ECE environments are essential when trying to intervene and change the ECE environment and ultimately influence child behaviors [20]. Instruments like the Environment and Policy Assessment and Observation (EPAO), with demonstrated reliability and validity, provide a fairly comprehensive assessment of the ECE environment [21, 22]. The EPAO captures multiple aspects of the nutrition and physical activity environment at child care, including foods and beverages served, opportunities for active play, provider-child interactions around food and activity, education for children and parents, professional development, and policies. An important limitation for existing tools, including the EPAO, is that most have been developed for use with center-based ECE programs.

As researchers begin to work within different ECE programs, such as FCCHs, existing measures will likely require modification. Given that FCCH providers take care of children in their own homes, this setting may pose specific challenges for those collecting data. For example, child care centers generally divide children into different classrooms by age, allowing teachers to more easily provide age-appropriate activities. In contrast, FCCH providers care for infants, toddlers, and preschoolers. These providers must provide activities that accommodate children at different developmental stages simultaneously. FCCH providers create a home-like environment for children they care for, which tends to be less formal with fewer written policies.

We address this gap in the current study by modify the EPAO for use in FCCHs and evaluating the reliability and validity of this new instrument. Specifically, we describe the modifications made to the EPAO and justifications for these changes. We also describe the methods used for and results from reliability and validity testing with FCCH providers.

## Methods

Modification of the EPAO was completed as part of a larger randomized-control trial evaluating a FCCH-based intervention, Keys to Healthy Child Care Homes (Keys) [6]. The goal of the intervention was to improve FCCH environments (provisions, practices, and policies) related to nutrition and physical activity to foster healthy habits in young children. The Keys study provided the opportunity to modify the EPAO for use in FCCHs and to examine this new protocol’s reliability and validity. All study protocols were reviewed by the Institutional Review Boards at the University of North Carolina at Chapel Hill and Duke University Medical Center. Protocols are also registered at ClinicalTrials.gov (NCT01814215, March 15, 2013).

### Measure modifications

The EPAO is designed to assess nutrition and physical activity environments of child care centers using a combination of direct observation and review of formal documents (e.g., policies and procedures manuals, training certifications), both of which are completed by specially trained and certified data collectors [21]. The original EPAO, developed in 2006, includes 192 items (102 from the observation and 90 from the document review) and is used to assess 8 nutrition and 8 physical activity environmental components [21], and 51 nutrition and 24 physical activity best practices [23]. At the time of its original development, the EPAO represented one of the most comprehensive assessments for nutrition and physical activity environments of ECE programs, and was therefore selected for use in the Keys intervention study. This study offered us the opportunity to incorporate improvements, address limitations, and to create a version that was tailored for use with FCCHs. Changes to the EPAO for use in FCCHs include restructuring of data collection across the day, broadening of its scope, and tailoring to the family child care setting. These changes are described below.

#### Restructuring data collection

One improvement that was carried forward from our development of a self-report version of the EPAO [18] was the use of a diary-type format. To make it easier for staff to report on daily activities, forms recorded information about various activities as they occurred temporally across the day. For example, the original EPAO included a single item that asked observers how many times fruit was served that day. Data collectors using the original tool had to make note of this information and then summarize it for the observation form. By adopting this restructured format, data collectors could capture detailed information about each meal and snack as it occurred, and then use this information to calculate daily totals later. Reformatting helped reduce the potential for data collector error; therefore, this restructured format was retained for the modification of the EPAO for FCCHs.

#### Broadening scope

New scientific literature and national guidelines had been published since the EPAO’s original development; hence the tool was no longer providing a comprehensive assessment of best practices [24]. In the area of child nutrition, new best practices emerged for limiting high-fat, high-salt, and high-sugar foods, avoiding flavored milk, avoiding the use of pressure and bribes, and having teachers enthusiastically role model and praise eating healthy foods. Child physical activity best practices also expanded to recognize the importance of outdoor play and screen time. In order to ensure that the EPAO remained a comprehensive assessment, new items were incorporated to assess compliance with these emerging best practices. Additional file 1: Table S1 describes the revisions made to content resulting from this broadening scope.

#### Tailoring to the FCCH

Tailoring of the EPAO required familiarity with how FCCH programs are typically structured and managed. To build familiarity with this setting, data collectors completed the EPAO (restructured format) in a small pilot involving four FCCHs. Data collectors and an EPAO training specialist (research assistant with experience collecting EPAO data and training other research teams on EPAO protocols) debriefed after each administration to refine definitions within the training manual. In addition, two EPAO training specialists conducted half-day observations in two FCCHs. Observations were unstructured and only qualitative impressions about the environment were collected. This pilot testing quickly identified key differences between center-based and FCCH-based programs related to staffing, space, and structure, which influenced item tailoring and scoring.

In terms of *staffing* in FCCHs, a single provider is often caring for children of multiple ages at the same time, and there are certain times of day when they are trying to manage multiple responsibilities. Provider-child interactions look quite different depending on the age of the child (e.g., infant, toddler, preschooler). For example, toddlers and preschoolers benefit from providers modeling healthy eating, offering praise and verbal encouragement, and avoiding the use of food bribes. However, some of these feeding interactions are not appropriate for use with infants. Hence, feeding and physical activity interactions for different ages of children must be assessed separately. Since the modification of the EPAO was being undertaken as part of a study with children 1.5–5 years old, assessment focused on provider interactions with toddlers and preschoolers. While infants may be cared for in these FCCHs, provider-infant interactions and infant-specific practices (e.g., breastfeeding, tummy time) were not assessed. In addition to caring for multiple ages of children, there are certain times of day that providers are also managing multiple responsibilities. Providers rarely have additional support staff, so at meal and snack times they must balance responsibilities of watching children and preparing food. Best practices must take such constraints into consideration. Hence, the original center-based scoring of certain items was adjusted for use in FCCHs to allow slightly more leniency.

In terms of *space*, FCCHs had greater variation in the amount of space available. Smaller FCCHs may convert the living room into the child care area, while larger FCCHs may have a separate entrance to a dedicated space with its own kitchenette and bathroom. However, the eating space and play space are often the same or at least connected. The outdoor play space is often limited and there are fewer play areas and portable play equipment. These space limitations were considered, particularly when scoring.

In terms of *structure*, FCCHs generally have a more flexible schedule to accommodate the staffing and space constraints noted above. For example, FCCHs generally have indoor playtime instead of center time because there is limited space for designated activity centers. The use of active screen time appeared more common, where the provider organizes activities around dancing or yoga videos.

The improvements and modifications were incorporated into the restructured format to create the EPAO for FCCHs. Training and certification protocols for data collectors were also updated accordingly. These changes also necessitated the creation of a scoring rubric (described below).

### Scoring rubric

A scoring rubric was developed to facilitate summarization of these data. The first step involves summing similar items from across the day to determine daily totals. For example, the EPAO uses time stamps to capture the specific start and stop times for periods of active play during activities such as indoor playtime, circle time, teacher-led activities, and outdoor playtime. These time stamps are used to calculate the length of each activity (end time minus start time). Active playtime is calculated for each of the morning and afternoon activities (counting any occasions where children had at least a light level of activity), and then summed across the entire day to determine the total active playtime. In the second step, daily totals are used to assess compliance with best practices. For each of the 38 nutrition and 27 physical activity best practices, compliance is rated on a scale of 0 to 3 with higher ratings indicating closer compliance. For example, if the best practices is to provide 90 min or more of active playtime per day, then FCCHs that provide less than 60 min receive a score of 0, those that provide 60–74 min score a 1, those that provide 75–89 min score a 2, and those that provide 90 min or more receive a score of 3. This scoring is similar to the original scoring of the EPAO, but uses 4-point scale instead of a 3-point scale.

The scoring rubric then calculates 7 nutrition-related and 10 physical activity-related environmental sub-scores. These environmental sub-scores are created by averaging scores from similar best practice compliance variables. For example, the environmental sub-score for time provided averages the scores from three best practice compliance variables – providing 90 min or more of active play, providing 45 min or more of teacher-led activity, and limiting bouts of seated time to less than 15 min. These environmental sub- scores can then be used to calculate overall nutrition and physical activity scores by summing all of the environmental summary scores. Table 1 presents a summary of this scoring rubric and the variables calculated throughout the process.

**Table 1:** EPAO for FCCH scoring rubric based on best practice^a^ (BP) compliance

Overall score component	Environmental sub-scores (# BP compliance items)	Example best practices
Overall nutrition score Range: 0–21	Seven nutrition-related environmental sub-scores	
	• Food provided (12 BPs)	• Fruit (not juice) served ≥2 times per day
	• Beverages provided (5 BPs)	• 100% fruit juice served ≤2 times per week
	• Feeding environment (7 BPs)	• Children served food family style
	• Feeding practices (8 BPs)	• Teachers always praise children for trying new or less preferred foods
	• Menus and variety (1 BP)	• Menu cycle is ≥3 weeks
	• Nutrition education and professional development (4 BPs)	• Children participate in planned nutrition education ≥1 time per week
	• Nutrition policy (1 BP)	• There is a comprehensive, written nutrition policy
Overall physical activity score Range: 0–30	Ten physical activity-related environmental sub-scores	
	• Time provided for physical activity (3 BPs)	• Preschool children are provided with ≥120 min per day of physical activity
	• Indoor play equipment (2 BPs)	• A large variety of equipment is available and in good condition to use indoors
	• Physical activity practices (3 BPs)	• Teachers never take away physical activity for longer than 5 min as a way of managing child behaviors
	• Physical activity education and professional development (4 BPs)	• Teachers and staff receive professional development of child physical activity ≥2 times per year
	• Physical activity policy (1 BP)	• There is a comprehensive, written physical activity policy
	• Time provided for outdoor play (2 BPs)	• Preschool children and toddlers get outside playtime ≥3 times per day
	• Outdoor play environment (5 BPs)	• Portable play equipment is always available during outdoor playtime
	• Screen time (4 BPs)	• No TV present or stored outside of classroom
	• Screen time practices (2 BPs)	• Screen time is rarely/never used as a reward
	• Screen time policy (1 BP)	• There is a comprehensive, written screen time policy

^a^Best Practice (BP) compliance is based on a series of evidence-based, or evidence-informed nutrition and physical activity practices associated with healthy weight development in preschool-aged children

### Data collection

Data for this study drew from a larger randomized control trial that was evaluating the impact of the Keys intervention in a sample of 166 FCCHs. This modified EPAO protocol was incorporated into the Keys intervention study to evaluate the impact on FCCH nutrition and physical activity environments. Per the protocol for the larger Keys study, a two-day measurement visit was conducted with all participating FCCHs (*n* = 166) at baseline and post-intervention (about 9 months apart). This two-day visit was spread across three days (e.g., Monday and Wednesday, Tuesday and Thursday). Data collectors collected EPAO observation data on both days, and requested necessary documents from the FCCH provider to complete the document review.

In addition to environmental data, children’s dietary intake and physical activity were also assessed during these measurement visits. Data were only collected from 1.5–5-year-old children with parental consent to participate only, which generally involved 2–4 children per FCCH. To capture dietary intake, all meals and snacks eaten by participating children while at child care were captured using a standard diet observation for child care protocol [25]. Diet observations were collected during both days of the visit. Diet observation data for foods consumed was entered into the Nutrition Data System for Research (NDSR) software (University of Minnesota, Minneapolis MN, 2014), which then applies its database of foods to estimate intakes of energy (kcals), macro- and micronutrients, and servings of different food groups. These data were then used to calculate Healthy Eating Index (HEI) scores [26], which provides an assessment of diet quality based on 2010 national dietary guidelines. To capture physical activity, participating children (1.5–5 years old) were fit with an accelerometer belt on the morning of the first day, which they then wore for the next three days (during waking hours). Belts contained an ActiGraph GT3X+ accelerometer (ActiGraph LLC, Pensacola FL) programmed to collect data in 40 Hz. Raw data were downloaded and summarized in 5 s epochs. Accelerometer data were isolated for the child care day; and only children with at least 2.5 h per day of wear at child care were retained. Age-appropriate cut-points are then applied to calculate children’s minutes per hour of moderate to vigorous physical activity (MVPA, counts ≥191.3 per 5 s) [27].

For reliability testing, we assessed inter-rater agreement between two data collectors assessing the same FCCH on the same day. To capture the additional data needed for this testing, two data collectors were sent into a sub-sample of 32 FCCHs to collect EPAO observation data independently. Data collectors were not asked to duplicate the document review since the availability of the required documents is often limited in FCCHs. This sub-sample was selected based on convenience, using FCCHs where more than three children were participating. In these FCCHs, two data collectors were needed to complete the diet observations (as the protocol specifies that each data collector cannot assess more than three children at a time [25]). The reliability data were collected at either the baseline or post-intervention time point; however, data collectors were always blinded. In total, we collected EPAO observation data on 61 different days completed by two data collectors (29 FCCHs had 2 days of reliability data, 3 FCCHs had only 1 day of data). EPAO observation data from each data collector from each day was scored separately (see EPAO scoring described above).

For construct validity testing, we used Key’s baseline data from all 166 FCCHs to assess associations between environmental characteristics and children’s dietary and physical activity behaviors. For this analysis, we first calculated average HEI score and average minutes per hour of MVPA for each FCCH. These home-level estimates of child diet quality and physical activity were then used to calculate correlations with EPAO scores. We were able to use the baseline data that was collected on 166 FCCH and the 496 participating children (495 with adequate diet data and 479 with adequate physical activity data).

### Statistical analyses

Descriptive statistics were calculated for each of analytic samples of FCCHs – reliability testing (*n* = 32) and validity testing (*n* = 166). For each, we examined demographic characteristics as well as means and standard deviations of overall nutrition and physical activity scores and the 17 related environmental sub-scores.

To examine reliability, we calculated intraclass correlations (ICC) between data collectors assessing the same home on the same day for each of the environmental sub-scores. Interpretation of ICC scores was based on Cicchetti’s recommendations in which ICC of <0.40 is poor, 0.40–0.59 is fair, 0.60–0.74 is good, and 0.75–1.00 is excellent [28]. In addition, we calculated percent agreement and kappas (simple and weighted, where appropriate) for each of the best practice compliance variables. Interpretation of percent agreement is based on Nuendorf’s recommendations, in which 0.90 is nearly always acceptable, 0.80 or greater is acceptable in most situations, and 0.70 may be appropriate in some exploratory studies [29]. Interpretation of kappas was based on McHugh’s recommendations, in which kappa of 0–0.20 indicates no agreement, 0.21–0.39 indicates minimal agreement, 0.40–0.59 indicates weak agreement, 0.60–0.79 indicates moderate agreement, 0.80–0.90 indicates strong agreement, and above 0.90 indicates almost perfect agreement [30]. In these analyses, not all environmental sub-scores could be calculated since some depended completely on document review data which was not collected twice as part of reliability testing.

For construct validity testing, we calculated correlations between EPAO environmental sub-scores and a FFCH average child HEI score or average minutes per hour of MVPA (depending on whether environmental sub-score was related to nutrition or physical activity). Two correlation matrices were also developed to examine associations between environmental sub-scores related to nutrition or physical activity. All analyses were performed using SAS version 9.4 (SAS Institute, Inc., Cary, NC).

## Results

Table 2 presents the demographic characteristics and EPAO scores of the FCCHs used for reliability and construct validity testing. Across both samples, nearly all FCCHs participated in the Child and Adult Care Food Program (CACFP), which is a federally funded program that reimburses participating child care programs for providing meals and snacks served to low-income children. The providers running these FCCHs were all female with an average age of 49 years old, and the majority was African American.

**Table 2:** Descriptive characteristics of FCCHs in reliability and construct validity samples

	Total sample *n* = 166	Reliability sample *n* = 32
FCCH Programs		
Star Rating^a^		
1 or 2 stars	13 (7.8%)	3 (9.4%)
3 stars	40 (24.1%)	13 (40.6%)
4 stars	68 (41.0%)	8 (25.0%)
5 stars	45 (27.1%)	8 (25.0%)
Accepts CACFP^b^ Subsidy	151 (91.0%)	31 (96.9%)
FCCH Providers		
Age (years)	49.3	49.1
Race		
Black or African American	123 (74.1%)	22 (68.8%)
White	30 (18.1%)	8 (25.0%)
Other	13 (7.8%)	2 (6.2%)
Hispanic or Latino Ethnicity	8 (4.8%)	0 (0%)
Education		
High school diploma or GED	41 (24.7%)	7 (21.9%)
Associate’s degree or 60 h college credit	82 (49.4%)	18 (56.2%)
Bachelor’s degree+	42 (25.3%)	7 (21.9%)

^a^Star Rating is a program that assesses the quality of the child care program. Ratings can range between 1 and 5 stars, with more stars equating to higher quality care

^b^CACFP refers to the Child and Adult Care Food Program, a federally funded program that reimburses participating child care programs for providing meals and snacks served to low-income children

As shown in Table 3, ICCs for overall nutrition and physical activity scores and the related environmental sub-scores generally demonstrated good-to-excellent inter-rater agreement. Of the 13 environmental sub-scores calculated in this analysis, seven demonstrated excellent agreement (ICCs above 0.75) and four demonstrated good agreement (ICC between 0.60 and 0.74). Only one environmental sub-score from nutrition and one from physical activity had ICC indicating fair or poor agreement. Four of the environmental sub-scores could not be calculated as they were derived from the document review and this analysis focused solely on data from the observation.

**Table 3:** Intraclass correlations assessing inter-rater agreement between data collectors observing the same FCCH on the same day

Environmental sub-score variable	ICC
Overall nutrition environment^a^	0.86
Foods provided	0.91
Beverages provided	0.96
Feeding environment	0.87
Feeding practices	0.56
Nutrition education and professional development	0.62
Overall physical activity environment^b^	0.76
Time provided for physical activity	0.83
Indoor play equipment	0.81
Daily physical activity practices	0.69
Physical activity education and professional development	0.22
Time provided for outdoor play	0.99
Outdoor play environment	0.84
Screen time	0.68
Daily screen time practices	0.67

^a^Environmental sub-scores for menus and variety and nutrition policy are missing as they depend solely on data from the document review. The overall nutrition score represents a sum of the remaining components

^b^Environmental sub-scores for physical activity policy and screen time policy are missing as they depend solely on data from the document review. The overall physical activity score represents a sum of the remaining components

Looking at inter-rater agreement for best practice compliance scores provides a more detailed examination, particularly for those environmental sub-scores with lower ICCs (see Additional file 2: Table S2 and Additional file 3: Table S3). In nutrition, 23 of the 34 best practice compliance scores had kappas greater than 0.60, thus indicating moderate or better agreement. Most of those with lower kappas did show good percent agreement, suggesting that the low kappas were the result of low variability in scores (e.g., food service style, provider eats healthy foods in front of children). Regarding physical activity, 14 of the 23 best practice compliance variables demonstrated good to excellent inter-rater agreement with kappas greater than 0.60 (see Additional file 2: Table S2 and Additional file 3: Table S3). Of the 9 remaining variables, it was not possible to calculate kappa for 2, but both had percent agreement above 90%.

Construct validity testing showed that many of the environmental sub-scores were related to children’s HEI scores or minutes per hour of MVPA (Tables 4 and 5). In terms of the nutrition environment, significant associations were observed between children’s HEI scores and overall nutrition environment score (*r* = 0.23, *p* = 0.003), as well as foods provided (*r* = 0.28, *p* = 0.0002), nutrition education and professional development (0.21, *p* = 0.007), and nutrition policy (*r* = 0.18, *p* = 0.022). There appeared to be some association between children’s diet and beverages provided (*r* = 0.15, *p* = 0.0504), but the association was not statistically significant. In terms of the physical activity environment, significant associations were seen between children’s MVPA and time provided for physical activity (*r* = 0.18, *p* = 0.023) and time provided for outdoor play (*r* = 0.20, *p* = 0.012). Unexpectedly, significant negative association were observed between children’s physical activity and screen time (*r* = −0.16, *p* = 0.042) and screen time practices (*r* = −0.21, *p* < 0.006).

**Table 4:** Correlation matrix between Child Diet Quality and EPAO Nutrition Scores (Pearson correlations and *p*-values)

	Overall nutrition envr^a^	Foods provided	Beverages provided	Feeding environment	Feeding practices	Menus	Nutrition educ and prof dev^b^	Nutrition policy
Child HEI	**0.23** ^*^ **0.0034**	**0.28** ^*^ **0.0002**	0.15 0.0504	0.05 0.5425	0.07 0.3942	0.05 0.4950	**0.21** ^*^ **0.0068**	**0.18** ^*^ **0.0223**
*Nutrition environment sub-components*								
1. Foods provided		1	0.12 0.1275	**0.15** ^*^ **0.0494**	0.08 0.2872	−0.02 0.8481	0.11 0.1614	0.12 0.1093
2. Beverages provided			1	**0.28** ^*^ **0.0002**	**0.19** ^*^ **0.0156**	−0.08 0.3007	0.04 0.6400	**0.26** ^*^ **0.0008**
3. Feeding environment				1	**0.27** ^*^ **0.0003**	0.02 0.7776	0.09 0.2615	0.10 0.1814
4. Feeding practices					1	0.05 0.5494	**0.30** ^*^ **<0.0001**	0.13 0.0975
5. Menus						1	0.05 0.5608	0.09 0.2665
6. Nutrition educ and prof dev^b^							1	**0.25** ^*^ **0.0010**
7. Nutrition policy								1

^a^Overall nutrition envr = overall nutrition environment

^b^Nutrition educ and prof deve = nutrition education and professional development

* The use of bold text with this asterik indicates correlations that were statistically significant (*p*<0.05)

**Table 5:** Correlation matrix between Child Physical Activity and EPAO Physical Activity Scores (Pearson correlations and *p*-values)

	Overall PA envr^a^	PA time provided^b^	Indoor play equip^c^	PA practices^d^	Screen time	ST practices^e^	Outdoor playtime	Outdoor play envr^f^	PA educ and prof dev^g^	PA policy^h^	ST policy^i^
Child MVPA	0.002 0.9826	**0.18** ^*^ **0.0228**	0.11 0.1549	0.04 0.5771	**−0.16** ^*^ **0.0419**	**−0.21** ^*^ **0.0060**	**0.20** ^*^ **0.0115**	0.04 0.5738	−0.04 0.6028	−0.13 0.0849	−0.03 0.6639
*Physical activity environment sub-components*											
1. PA time provided^b^		1	**0.19** **0.0126**	0.13 0.0955	−0.02 0.7718	**−0.18** ^*^ **0.0187**	**0.41** ^*^ **<0.0001**	**0.18** ^*^ **0.0237**	−0.05 0.5591	−0.07 0.3656	0.04 0.5838
2. Indoor play equip^c^			1	**0.34** ^*^ **<0.0001**	−0.10 0.2141	**−0.05** ^*^ **0.0187**	**0.29** ^*^ **0.0001**	**0.63** ^*^ **<0.0001**	**0.23** ^*^ **0.0024**	0.01 0.9173	−0.03 0.6928
3. PA practices^d^				1	0.03 0.7311	−0.01 0.8520	**0.27** ^*^ **0.0004**	**0.30** ^*^ **<0.0001**	**0.20** ^*^ **0.0024**	0.12 0.1264	0.09 0.2562
4. Screen time					1	**0.70** ^*^ **<0.0001**	−0.06 0.4148	−0.15 0.0623	0.01 0.8676	0.01 0.8652	0.02 0.7775
5. ST practices^e^						1	−0.15 0.0545	−0.08 0.3211	0.02 0.8066	0.15 0.0579	0.02 0.8401
6. Outdoor playtime							1	**0.45** ^*^ **<0.0001**	0.01 0.9126	−0.08 0.3125	0.10 0.2008
7. Outdoor play envr^f^								1	0.12 0.1349	−0.05 0.5170	−0.03 0.7126
8. PA educ and prof dev^g^									1	**0.18** ^*^ **0.0239**	0.10 0.2054
9. PA policy^h^										1	**0.41** ^*^ **<0.0001**
10. ST policy^i^											1

^a^Overall PA envr = overall physical activity environment

^b^PA time provided = physical activity time provided

^c^Indoor play equip = indoor play equipment

^d^PA practices = physical activity practices

^e^ST practices = screen time practices

^f^Outdoor play envr = outdoor play environment

^g^PA educ and prof dev = physical activity education and professional development

^h^PA policy = physical activity policy

^i^ST policy = screen time policy

* The use of bold text with this asterik indicates correlations that were statistically significant (*p*<0.05)

Correlation matrices examining associations between the environmental summary scores show some significant relationships (Tables 4 and 5). While several correlations between environmental sub-scores were statistically significant, the correlations were generally modest (often less than 0.30), indicating that they still capture unique aspects of the environment. Among the physical activity environmental sub-scores, two notably higher correlations were observed between indoor and outdoor play equipment (*r* = 0.63) and between screen time and screen time practices (*r* = 0.70). However, neither correlations is so high that it would indicate that these are measures are redundant.

## Discussion

This modification of the EPAO for use in FCCHs fills a gap among measurement tools essential for researchers interested in studying how ECE environments shape children’s eating and physical activity habits. The number of ECE-based interventions is rapidly increasing [31], and the EPAO has been a useful tool particularly for those evaluating center-based interventions [32–35]. There is a growing number of studies conducted in FCCHs and their unique characteristics present certain data collection challenges. Therefore, measurement tools that are reliable and valid within this setting are needed. The EPAO for FCCHs provides such an instrument and results of this study demonstrate its psychometric strengths.

A thorough process was used to develop the EPAO for FCCHs. Rigorous measures development should include a clear conceptualization of purpose and constructs to be measured, a systematic process for item pool generation and refinement, and thorough psychometric assessment including reliability, validity, and sensitivity to change testing [36–38]. The modification of the EPAO addressed many of these recommended elements except for sensitivity testing. Similar to the original, the EPAO for FCCH was conceptualized as a comprehensive assessment of FCCHs’ provisions, practices, and policies around nutrition and physical activity. While the original EPAO was used as a starting point for the item pool, new items were added to ensure that it captured all known best practices. Also, items were modified, pilot tested, and refined to make sure that they were appropriate for use in FCCHs. Results from the psychometric testing showed strong inter-rater reliability and good construct validity.

There were a couple of specific environmental sub-scores with unexpected reliability results. In nutrition, feeding practices had an overall ICC of 0.56; and 6 of the 8 best practice compliance variables going into that sub-score had kappas under 0.60. The lower reliability may have been due to the difficulty in measuring providers’ feeding practices. Data collectors were also doing observation of children’s dietary intakes during meals, which may make it more difficult to capture all of the provider-child interactions during these eating occasions. In physical activity, education and professional development had an ICCs of 0.22. However, there were only two best practice compliance variables within this section. Offering planned gross motor activities had both a low kappa (0.305) and percent agreement (67.2). However, the other item on informal physical activity education had a low kappa (0.115) but a high percent agreement (83.6), with lack of variation in responses driving the kappa down.

During construct validity testing, we also had unexpected findings. Less than half of the environmental sub-scores were associated with child nutrition or physical activity. The lack of significant associations may have been due in part to the lack of variability in sub-scores as many of these practices were used very infrequently. Additional development may also be needed for certain sections, such as feeding practices. While modifications made slight expansions to this section, it still assessed only a limited number of feeding practices. However, efforts are underway to expand on the breadth of feeding practices that the EPAO can be used to assess. In the physical activity section, we did not see a significant association between children’s MVPA and the overall physical activity score. In addition, we saw negative associations between children MVPA and screen time and screen time practices. These negative associations may speak to differences in how FCCHs use screens and may have contributed to the lack of association with the overall physical activity score. These findings suggest that use of screens in FCCHs warrants greater investigation in future research studies.

Another advancement offered by the EPAO for FCCHs is the development of a scoring rubric that allows the nutrition and physical activity environments to be summarized by two overarching scores (one for nutrition and one for physical activity). While the overarching scores are useful, the scoring rubric also calculates 17 environmental sub-scores (elements within the environment such foods provided, physical activity practices, nutrition and physical activity policy) as well as compliance with 67 best practice recommendations. In comparison, the original EPAO included 102 observation items and 90 document review items, which were collapsed into 16 environmental subscales [21]. The original EPAO scoring did not try to calculate overall nutrition and physical activity scores. The new scoring rubric should help address a key challenge of past assessment tools, providing a summary of the broader environment in addition to its sub-scores [37].

This study has many strengths including a rigorous development process and the use of high-quality measures of children’s diet (observation protocol) and physical activity (accelerometry) to evaluate construct validity; however, there are also some limitations to acknowledge. The current study did not assess the instrument’s internal validity (Cronbach’s alpha); however, it was deemed as an inappropriate assessment for this measure. The EPAO uses item level data to assess best practice compliance, each of which represents a unique construct. These best practice compliance variables are combined into sub-scores; however, the best practices components would not be expected to correlate with each other. For example, the feeding practices sub-score includes offering praise, assessing hunger/fullness before removing plates and before serving seconds, requiring children to remain seated during meals, using an authoritarian feeding style, bribing with food, managing child emotions and behavior with food, and providing prompts to drink water. Also, the study did not assess sensitivity to change; however, it’s inclusion in the larger Keys study will provide for this evaluation in the future. The scoring rubric did revisit the original scoring protocol changing it from a 3-point scale to a 4-point scale when assessing compliance with best practices. In a 3-point scale, where the lowest score equates to minimal practice and the highest score equates to best practice, it is likely that most ECE programs would fall in the middle. The use of a 4-point scale should thus should be more precise in categorizing programs and allow greater sensitivity to change. Furthermore, the EPAO for FCCH is currently being used in a randomized control trial, results of which should speak to its sensitivity. Another limitation is the lower psychometric performance of the sub-score related to feeding practices. Feeding practices, as it is currently assessed, may require more subjective evaluation by data collectors. Additional refinements in training would likely allow for more consistent assessment.

## Conclusions

The EPAO for the FCCH instrument is a useful tool for researchers working with this unique type of ECE setting. It has undergone rigorous development and testing and appears to have good psychometric properties. Assessment of providers’ feeding practices may benefit from additional training, which in turn may produce greater reliability between observers. Greater investigation into the use of screens in FCCHs is also need to understand how they are impacting children’s physical activity.

## Additional files

Additional file 1: Table S1. Comparison of content for sections in the original EPAO vs. EPAO for FCCH. (DOCX 13 kb)

Additional file 2: Table S2. Inter-rater agreement on nutrition best practices compliance items, observed range of values, percent agreement, and kappas. (DOCX 14 kb)

Additional file 3: Table S3. Inter-rater agreement on physical activity best practices compliance items, observed range of values, percent agreement, and kappas. (DOCX 14 kb)

## Abbreviations

BP: Best practices
CACFP: Child and adult care food program
ECE: Early Care and Education
EPAO: Environment and Policy Assessment and Observation
FCCH: Family child care home
HEI: Healthy Eating Index
ICC: Intraclass correlation
MVPA: Moderate to vigorous physical activity
NDSR: Nutrition Data System for Research
PA: Physical activity
ST: Screen time
