# Supplementary material for: Assessment of nutrition and physical activity environments in family child care homes: modification and psychometric testing of the Environment and Policy Assessment and Observation
Source: BMC Public Health. 2017 Aug 29;17:680. doi: 10.1186/s12889-017-4686-9 (PMC5576128; doi:10.1186/s12889-017-4686-9)
Supplement: Supplementary file 1 — Comparison of content for sections in the original EPAO vs. EPAO for FCCH. (DOCX 13 kb) [file 12889_2017_4686_MOESM1_ESM.docx]

**Table S1. Comparison of content for sections in the original EPAO vs. EPAO for FCCH**

| **Original EPAO** | **EPAO for FCCH** | **Description of Changes** |
| --- | --- | --- |
| ***Nutrition*** | ***Child Nutrition*** |  |
| Fruits and Vegetables  Grains and Low-fat Meats  High-fat/High-sugar Foods | Foods Provided | Items around foods provided were combined into a single category. One additional item was added to capture high-fat/high-salt snacks. |
| Beverages | Beverages Provided | One additional item was added to capture fruit juice. Scoring was also adjusted to incorporate flavored milk. |
| Staff Behavior Nutrition | Feeding Practices | Several items were added to capture feeding practices such as asking child about hunger/fullness before removing plate (in addition to before serving seconds), requiring children to sit at the table until others are finished eating, offering food as a bribe, and using an authoritative feeding style. |
| Nutrition Environment | Feeding Environment | Some of the items in the original staff behavior section were moved into feeding environment, such as sitting with children during meals. New items were also added to capture environmental characteristics such as having the presence of TV during mealtimes, teachers eating the same foods as children, and teachers enthusiastically modeling healthy eating. |
| Nutrition Training and Education | Nutrition Education and Professional Development | Items in the original staff behavior section about formal and informal child education were moved into this category. Items were revised to capture greater detail about the frequency and content of parent education and staff professional development. |
| Nutrition Policy | Nutrition Policy | Items were revised to capture greater detail regarding the content of nutrition policies and whether they fully or partially captured best practices. |
| ***Physical Activity*** | ***Child Physical Activity and Outdoor Play and Learning*** | Physical activity content was divided to better capture physical activity (generally indoors), outdoor play and learning, and screen time. |
| Active Opportunities | Time Provided for Physical Activity  Time Provided for Outdoor Play | Content of items was similar, but separated across two sections – one was specific to indoor play and the other to outdoor play. |
| Portable Play Equipment  Fixed Play Equipment | Indoor Play Equipment  Outdoor Play Environment | Instead of characterizing the physical environment based on presence of portable vs. fixed play equipment, it was defined more broadly and characterized according to what was present indoors vs. outdoors. The indoor play equipment sub-component includes portable play equipment indoors and posters/books promoting healthy eating. Items were revised to also capture presence of posters/ books promoting unhealthy eating. Outdoor play equipment includes portable and fixed equipment as well as new items about accessibility of equipment, teacher offering of equipment, presence of shaded play areas, and presence of a garden. |
| Staff Behavior Physical Activity | Daily Physical Activity Practices | Content of items was similar. |
| Physical Activity Training and Education | Physical Activity Education and Professional Development | Items in the original staff behavior section about formal and informal child education were moved into this category. Items were revised to capture greater detail about the frequency and content of parent education and staff professional development. |
| Physical Activity Policy | Physical Activity Policy | Items were revised to capture greater detail regarding the content of nutrition policies and whether they fully or partially captured best practices. |
|  | ***Screen Time*** |  |
| Sedentary Opportunities  Sedentary Environment | Screen Time | Content of items was expanded slightly to also capture whether TV and videos were educational and commercial free and whether an alternate activity was offered during screen time. |
|  | Daily Screen Time Practices | Items were added to capture practices such as the use of screen time as a reward and teachers engaging with children during screen time activities. |
|  | Screen Time Policy | Items were added to capture screen time policies specifically. |
