# Supplementary material for: Assessment of nutrition and physical activity environments in family child care homes: modification and psychometric testing of the Environment and Policy Assessment and Observation
Source: BMC Public Health. 2017 Aug 29;17:680. doi: 10.1186/s12889-017-4686-9 (PMC5576128; doi:10.1186/s12889-017-4686-9)
Supplement: Supplementary file 2 — Inter-rater agreement on nutrition best practices compliance items, observed range of values, percent agreement, and kappas. (DOCX 14 kb) [file 12889_2017_4686_MOESM2_ESM.docx]

**Table S2. Inter-rater agreement on nutrition best practices compliance items, observed range of values, percent agreement, and kappas**

| **Variable** | **Label** | **Obs. Range** | **% Agree** | **Kappa** | **95% CI** |
| --- | --- | --- | --- | --- | --- |
| ***Nutrition – Foods Provided*** | | | | | |
| Nutr_score_1 | Whole fruit | 2.0-3.0 | 98.4 | 0.924 | 0.758, 1.000 |
| Nutr_score_2 | Fresh, frozen, canned in juice | 0.0-3.0 | 90.2 | 0.903 | 0.813, 0.992 |
| Nutr_score_3 | Total vegetables | 0.0-3.0 | 88.5 | 0.832 | 0.682, 0.981 |
| Nutr_score_4 | Dark colored vegetables | 0.0-3.0 | 93.4 | 0.859 | 0.670, 1.000 |
| Nutr_score_5 | Vegetables w/out added fat | 0.0-3.0 | 98.4 | 0.971 | 0.909, 1.000 |
| Nutr_score_6 | Fried potatoes | 0.0-3.0 | 100.0 | 1.000 | 1.000, 1.000 |
| Nutr_score_7 | Fried meats | 0.0-3.0 | 96.7 | 0.837 | 0.595, 1.000 |
| Nutr_score_8 | High-fat meats | 0.0-3.0 | 95.1 | 0.887 | 0.755, 1.000 |
| Nutr_score_9 | Lean meats | 0.0-3.0 | 83.6 | 0.662 | 0.453, 0.870 |
| Nutr_score_10 | High-fiber, whole grains | 0.0-3.0 | 90.2 | 0.830 | 0.694, 0.967 |
| Nutr_score_11 | High-sugar and high-fat foods | 0.0-3.0 | 98.4 | 0.792 | 0.229, 1.000 |
| Nutr_score_12 | Sweet and salty snacks | 0.0-3.0 | 95.1 | 0.849 | 0.666, 1.000 |
| ***Nutrition – Beverages Provided*** | | | | | |
| Nutr_score_14 | Water availability | 0.0-3.0 | 90.2 | 0.760 | 0.530, 0.991 |
| Nutr_score_15 | Fruit juice | 0.0-3.0 | 88.5 | 0.788 | 0.634, 0.942 |
| Nutr_score_16 | Sugary drinks | 0.0-3.0 | 96.7 | 0.732 | 0.495, 0.970 |
| Nutr_score_17 | Milk type | 0.0-3.0 | 100.0 | 1.000 | 1.000, 1.000 |
| Nutr_score_18 | Flavored milk | 0.0-3.0 | 100.0 | 1.000 | 1.000, 1.000 |
| ***Nutrition – Feeding Environment*** | | | | | |
| Nutr_score_19 | Food service style | 2.0-3.0 | 96.7 | -0.017 | -0.064, 0.031 |
| Nutr_score_20 | TV on during meals | 0.0-3.0 | 88.5 | 0.765 | 0.590, 0.940 |
| Nutr_score_21 | Providers eats same food | 0.0-3.0 | 83.6 | 0.814 | 0.700, 0.928 |
| Nutr_score_22 | Provider eats unhealthy food | 1.0-3.0 | 85.2 | 0.593 | 0.345, 0.841 |
| Nutr_score_23 | Enthusiastic role modeling | 0.0-2.0 | 77.0 | 0.518 | 0.289, 0.747 |
| Nutr_score_24 | Healthy eating materials | 0.0-3.0 | 86.9 | 0.794 | 0.655, 0.934 |
| Nutr_score_25 | Unhealthy eating materials | 0.0-3.0 | 88.5 | 0.831 | 0.701, 0.961 |
| ***Nutrition – Feeding Practices*** | | | | | |
| Nutr_score_26 | Verbal praise | 0.0-3.0 | 60.6 | 0.370 | 0.190, 0.551 |
| Nutr_score_27 | Ask before removing plates | 0.0-3.0 | 44.3 | 0.349 | 0.110, 0.588 |
| Nutr_score_28 | Ask if hungry before seconds | 0.0-3.0 | 65.6 | 0.626 | 0.446, 0.806 |
| Nutr_score_29 | Sit at table until finished | 1.0-3.0 | 88.5 | 0.528 | 0.141, 0.915 |
| Nutr_score_30 | Authoritative feeding | 0.0-3.0 | 65.6 | 0.137 | -0.162, 0.435 |
| Nutr_score_31 | Food bribes | 0.0-3.0 | 75.4 | 0.406 | 0.164, 0.648 |
| Nutr_score_32 | Food as comfort | 0.0-3.0 | 75.4 | 0.406 | 0.164, 0.648 |
| Nutr_score_35 | Prompts to drink water | 0.0-3.0 | 80.3 | 0.798 | 0.698, 0.897 |
| ***Nutrition - Education*** | | | | | |
| Nutr_score_38 | Planned nutrition education | 0.0-3.0 | 96.7 | 0.483 | -0.549, 1.000 |
| Nutr_score_39 | Informal nutrition education | 0.0-2.0 | 67.2 | 0.435 | 0.259, 0.612 |
