# Supplementary material for: Assessment of nutrition and physical activity environments in family child care homes: modification and psychometric testing of the Environment and Policy Assessment and Observation
Source: BMC Public Health. 2017 Aug 29;17:680. doi: 10.1186/s12889-017-4686-9 (PMC5576128; doi:10.1186/s12889-017-4686-9)
Supplement: Supplementary file 3 — Inter-rater agreement on physical activity best practices compliance items, observed range of values, percent agreement, and kappas. (DOCX 14 kb) [file 12889_2017_4686_MOESM3_ESM.docx]

**Table S3. Inter-rater agreement on physical activity best practices compliance items, observed range of values, percent agreement, and kappas**

| **Variable** | **Sample** | **Range** | **% Agree** | **Kappa** | **95% CI** |
| --- | --- | --- | --- | --- | --- |
| ***Physical Activity – Time Provided*** | | | | | |
| PA_score_1 | Indoor and outdoor play time | 0.0-3.0 | 67.2 | 0.535 | 0.339, 0.730 |
| PA_score_3 | Provider-led physical activity | 0.0-3.0 | 82.0 | 0.740 | 0.570, 0.910 |
| PA_score_4 | Length of seated time | 0.0-3.0 | 82.0 | 0.778 | 0.622, 0.933 |
| ***Physical Activity – Indoor Play Environment*** | | | | | |
| PA_score_6 | Portable play equipment | 0.0-3.0 | 77.0 | 0.751 | 0.631, 0.872 |
| PA_score_9 | Posters and books promoting physical activity | 0.0-3.0 | 93.4 | 0.860 | 0.730, 0.990 |
| ***Physical Activity – Physical Activity Practices*** | | | | | |
| PA_score_10 | Withholding physical activity as punishment | 2.0-3.0 | 98.4 | 1.000 | 1.000, 1.000 |
| PA_score_11 | Provider role during active play | 0.0-3.0 | 67.2 | 0.620 | 0.472, 0.768 |
| PA_score_13 | Physical activity in routines and transitions | 0.0-2.0 | 63.9 | 0.215 | -0.010, 0.440 |
| ***Physical Activity - Education*** | | | | | |
| PA_score_14 | Planned gross motor lessons | 0.0-3.0 | 67.2 | 0.305 | 0.017, 0.594 |
| PA_score_15 | Informal physical activity education | 0.0-2.0 | 83.6 | 0.115 | -0.184, 0.413 |
| ***Outdoor Play – Time Provided*** | | | | | |
| OPL_score_1 | Occasions of outdoor play | 0.0-3.0 | 100.0 | 1.000 | 1.000, 1.000 |
| OPL_score_2 | Outdoor play time | 0.0-3.0 | 93.4 | 0.899 | 0.762, 1.000 |
| ***Outdoor Play – Play Environment*** | | | | | |
| OPL_score_4 | Shaded play space | 0.0-3.0 | 93.4 | 0.908 | 0.814, 1.000 |
| OPL_score_5 | Open play area | 0.0-3.0 | 96.7 | 0.967 | 0.918, 1.000 |
| OPL_score_7 | Garden | 0.0-3.0 | 98.4 | 1.000 | 1.000, 1.000 |
| OPL_score_9 | Offering portable play equipment | 0.0-2.0 | 90.2 | 0.820 | 0.644, 0.996 |
| OPL_score_10 | Accessibility of portable play equipment | 0.0-3.0 | 75.4 | 0.569 | 0.312, 0.827 |
| ***Screen Time - Availability*** | | | | | |
| ST_score_1 | Location of TVs | 0.0-3.0 | 78.7 | 0.730 | 0.544, 0.916 |
| ST_score_2 | TV Time | · or 3.0 | 91.8 | NA | NA |
| ***Screen Time – Teacher Practices*** | | | | | |
| ST_score_4 | Education and commercial free programming | 0.0-3.0 | 91.8 | 0.732 | 0.476, 0.988 |
| ST_score_5 | Alternate activity offered during screen time | 0.0-3.0 | 80.3 | 0.397 | -0.051, 0.845 |
| ST_score_6 | Screen time as reward | 3.0 | 100 | NA | NA |
| ST_score_7 | Teachers engaging with children during screen time | 0.0-3.0 | 77.0 | 0.508 | 0.285, 0.732 |

NA = Kappas could not be calculated because of no variation, either 3 or missing.
